# Gender norms, HIV risk, and attitudes towards pre-exposure prophylaxis and other HIV preventive interventions among South African adolescents

**DOI:** 10.11604/pamj.2022.41.136.32881

**Published:** 2022-02-16

**Authors:** Israel Agaku, Lungile Nkosi, Joy Gwar, Tina Tsafa

**Affiliations:** 1 Department of Oral Health Policy and Epidemiology, Harvard School of Dental Medicine, Boston, MA, United States,; 2 The Africa Center for Tobacco Industry Monitoring and Policy Research, Sefako Makgatho Health Sciences University (SMU), Pretoria, South Africa,; 3 Federal Medical Centre, Makurdi, Benue State, Nigeria,; 4 Department of Mass Communication, Benue State University, Makurdi, Nigeria

**Keywords:** PrEP, HIV, prevention, condom, adolescents

## Abstract

**Introduction:**

we examined HIV-related social norms and perceptions among South African adolescents aged 15-18 years and evaluated their openness to using pre-exposure prophylaxis (PrEP) to reduce HIV risk.

**Methods:**

we analyzed cross-sectional data of 4,567 adolescents aged 15-18 years from the “fifth South African National HIV prevalence, HIV incidence, behaviour and communication survey (2017/2018)”. Outcomes of interest in our study were HIV-related social norms and awareness of HIV prevention methods, including pre-exposure prophylaxis (PrEP).

**Results:**

our results showed that compared to their female counterparts, a significantly higher percentage of male adolescents endorsed the statement “Men can have two or more sexual partners at the same time” (14.2% vs 10.1%, p=0.021). Condoms were the most popular method of HIV prevention, with 83.5% of all participants reporting awareness. Yet, 35.4% of those sexually active in the past year reported not using condoms all the time. Perceived parental sex education and exposure to community campaigns for HIV prevention were both associated with increased awareness of HIV prevention measures and openness to PrEP. Unaided recall of PrEP was very low (3.7%), but most of those who were HIV seronegative (69.3%) were open to using it after learning about it. Openness towards PrEP was significantly higher among those reporting vs not reporting past-year sexual activity (adjusted prevalence ratio (APR=1.16, 95%CI, 1.06-1.28), and binge-drinking (APR=1.24, 95%CI, 1.08-1.41).

**Conclusion:**

this study showed that while many South African adolescents were interested in trying PrEP, initial awareness was low. Ensuring barrier-free access to evidence-based preventive strategies may benefit public health.

## Introduction

The HIV epidemic among the South African adolescent population differs from that in the adult population in risk factors, trends, and avertable trajectory. Evidence-based measures for prevention of mother-to-child transmissions such as providing antiretroviral therapy (ART) for mother and newborn, correct breastfeeding practices, and early child testing for HIV infection, averted an estimated 582,245 new HIV infections during 2003-2016 [1,2]. Yet, over half a million (578,192) new infections still occurred among South African children aged 0-14 years during that period-higher than any other country in the world [2,3]. The basis for concern about the large number of youths living with HIV is because of the high prevalence of teen risky sexual behaviors, pregnancies, low awareness of HIV prevention, as well as suboptimal ART adherence and retention in care. A study conducted in rural South Africa among adolescents aged 14-17 found that less than half of those sexually active reported a history of regular condom use (45.5% boys; 46.5% girls) [4]. Another study among South African adults aged 18-24 years in two districts with high HIV prevalence found that less than half (44.7%) had correct knowledge of HIV prevention [5]. For long-term impact, and especially among youth who are very impressionable, HIV prevention/control strategies must acknowledge and address the social norms that underpin HIV transmission. It is well known from the HIV and smoking epidemics, that knowledge alone is not sufficient for behavior change, social norms must be considered. Two types of social norms exist: descriptive (perceived prevalence), and injunctive social norms (perceived acceptability) [6,7]

Social norms in adolescence-the period between childhood and adulthood may affect and be affected by norms in the adult population because adolescents emulate authority figures such as parents, teachers, and other community role models [8]. Prevailing adult behaviors and norms, including gender norms, may therefore be adopted, and reinforced early in adolescence, and carried on into adulthood, but this has not been empirically evaluated in South Africa. With the 95-95-95 framework [9] focused solely on clinical, not social endpoints (increasing diagnosis, treatment, and viral suppression), and in the absence of youth-specific, socially-oriented benchmarks, measuring youth-related social norms can be challenging, but is nonetheless warranted. From a health equity perspective, a better understanding of differences in social norms and gender roles perceived by boys and girls in adolescence may provide insights into the wide inequalities in HIV prevalence between men and women in adulthood. The bold target to eradicate AIDS as a public health threat by 2030 [10], makes it imperative to understand gaps and opportunities for expanding prevention at all levels, including primary (e.g. population-level HIV prevention campaigns), secondary (e.g. use of pre-exposure prophylaxis or PrEP, among individuals at high risk for HIV), and tertiary (e.g. treatment as prevention among those diagnosed of HIV). To provide data that could inform youth-oriented HIV prevention programs in South Africa, this study was conducted among a nationally representative sample of South African adolescents aged 15-18 years, with three objectives; i) Investigate perceived norms along the intersection of gender, sexual intimacy, and power dynamics; ii) examine awareness and receptivity towards HIV prevention measures, including PrEP; iii) evaluate how perceived norms may be influenced by proximal social influences, including family, teachers, and the broader community.

## Methods

**Data collection:** we analyzed the fifth wave of the South African National HIV Prevalence, HIV Incidence, Behavior and Communication Survey (SABSSM V), a nationally representative, face-to-face, household survey of the South African civilian population [11]. All individuals living in a household (including hostels) were eligible (response rate=82.2%). The survey had both an interview (self-reported measures, e.g. perceived social norms and demographic information) and a laboratory component (biomarkers, e.g. HIV serostatus). Consent was provided by all adult respondents, and parental consent was obtained for minors ≤ 17 years.

### Measures

**HIV status and HIV perceived risk:** HIV status was assessed in both the interview (self-reported HIV status) and laboratory (objectively ascertained HIV status) components of the survey. Among HIV negative individuals or those with unknown status, the survey asked, “On a scale of 1 to 4 (with 1 being low and 4 being high), how would you rate yourself in terms of risk of becoming infected with HIV?” scores of ≥ 3 were classified as high perceived risk.

**Social norms related to HIV, gender, and sexual intimacy:** participants responded to several statements that started with the stem, “at your school, how often do? “boys sexually harass girls by touching, threatening, or making rude remarks to them?”; “girls sexually harass boys by touching, threatening, or making rude remarks to them?” “male educators propose relationships with girl pupils?” “female educators propose relationships with boy pupils?” and “teachers propose relationships with pupils of the same sex?” categorical response options of “always”/“often”/“sometimes” (vs “never”/ “don´t know”) were classified as affirmative answers. An affirmative response to any of the latter three educator/teacher-related indicators was classified to indicate exposure to a school environment where teachers have sexual relationships with students. Participants also indicated their agreement with the following gender-specific statements: “it is okay for young men/young women to have children before they are married”; “young women/men can have older male/female sexual partners for money, other necessities, or luxuries”; “women/Men can have two or more sexual partners at the same time”.

**Awareness of PrEP and other HIV preventive measures:** the survey assessed participants´ knowledge that “a healthy-looking person can have HIV. Awareness of HIV preventive measures was assessed using both unaided and aided recall. Unaided recall, also known as spontaneous recall, evaluates how well an individual can remember what is being assessed without the help of any external cues or clues. First, unaided recall was assessed with the question, “can you tell me all the ways you know how to prevent HIV infection?” next, for aided recall, a list of preventive measures was read to participants and for each measure, they could answer “yes” or “no” whether they believed it could prevent HIV. With novel medicines such as PrEP now available for HIV prevention, we were interested in exploring awareness (based on unaided recall) and interest (after describing the medicine). Interest in PrEP was assessed thus: “scientists are now studying a medication where, if taken orally every day, can reduce a person´s chances of getting HIV infection. If such a medication was available, would you want to take it?”

**Proximal social influences at home, school, and the community:** a proxy for parental sex education was an affirmative response (“agree” vs “disagree”/“don´t know”) to either statement below “parents are talking to their children about sex and HIV prevention”; “parents are encouraging their children to use condoms”. While these questions were not asked specifically in relation to the respondent´s parents, we assumed that an affirmative response would be more likely than not if the respondent received such education from their own parents. Participants were also asked whether they had “enough community-based organizations helping with HIV/AIDS in their community” and their extent of awareness or engagement with various community programs, campaigns, or other mass media messages for HIV prevention. The survey listed several HIV prevention campaigns in South Africa. Awareness of any HIV prevention campaign was defined as brand recognition of ≥1 campaign´s logo, or self-reported exposure to ≥1 campaign through any print or non-print media, including radio, television, newspaper, magazine, pamphlet, or social media. Engagement was defined as a report of being actively involved with a campaign, such as being a member of an HIV prevention club, participating in a dialogue on HIV-related issues, attending a workshop, community meeting, other educational event, or a clinic discussion in the community around HIV issues. Using these data, we created a categorical variable with three levels: individuals not aware of any campaign (i.e. campaign-unaware), individuals reporting only awareness but no engagement (i.e. campaign-aware), and those reporting past engagement (i.e. campaign-engaged).

**Other clinical, behavioral, and demographic characteristics:** data were collected on ever and past-year sexual activity, circumcision (for males), binge-drinking (having ≥5 drinks for males or ≥4 drinks for females on the same occasion for ≥1 day in the past month), and ever use of any illicit substance (e.g. cocaine). History of regular condom use was defined as a response of “every time” to the question “how often do you use a condom with your partner?” this was assessed for the three most recent sexual partners, and those who answered “every time” in relation to their current or previous sexual partners were defined as having reported a history of regular condom use. Demographic characteristics include gender, province, sexual orientation, race, and orphan hood status.

**Statistical analysis:** weighted percentages were calculated to characterize social norms and quantify awareness of various HIV preventive measures. Comparisons were performed with Chi-squared tests (p<0.05). To contrast factors associated with HIV seropositivity (among all participants) vs perceived HIV risk and openness towards PrEP (among seronegative participants), we calculated adjusted prevalence ratios in a Poisson regression model adjusted for age and gender. To measure associations between various social influences and HIV knowledge and perceptions, adjusted prevalence ratios were computed using pooled data of HIV seropositive and negative adolescents to increase sample size, adjusting for HIV status, geographic location, race, and sex. The social influences of interest (predictor variables) were perceived parental sex education at home, teachers´ sex behaviors at school, and HIV education campaigns in the community. Outcomes were awareness that “a healthy-looking person can have HIV” and awareness of various evidence-based HIV prevention measures (based on aided recall). Another model was fitted only among seronegative individuals to measure associations between social influences and openness to PrEP, adjusting for geographic location, race, and sex.

**Ethics approval:** this study was performed in line with the principles of the Declaration of Helsinki. This study was deemed non-human subject research as all data were publicly available, disidentified, secondary datasets. Ethical approval was therefore not sought.

**Consent to participate publish:** informed consent in the original survey was obtained from all individual participants included in the study.

## Results

**Characteristics of the study population:** of South African adolescents aged 15-18 years, 83.4% were black, 49.0% were female while 51.0% were males. Of males, 68.1% were circumcised. By province, distributions were KwaZulu-Natal (23.1%), Gauteng (21.7%), Limpopo (12.2%), Western Cape (10.6%), Eastern Cape (10.0%), Mpumalanga (8.9%), North-West (6.2%), Free State (5.2%), and Northern (2.2%).

**Figure 1:**
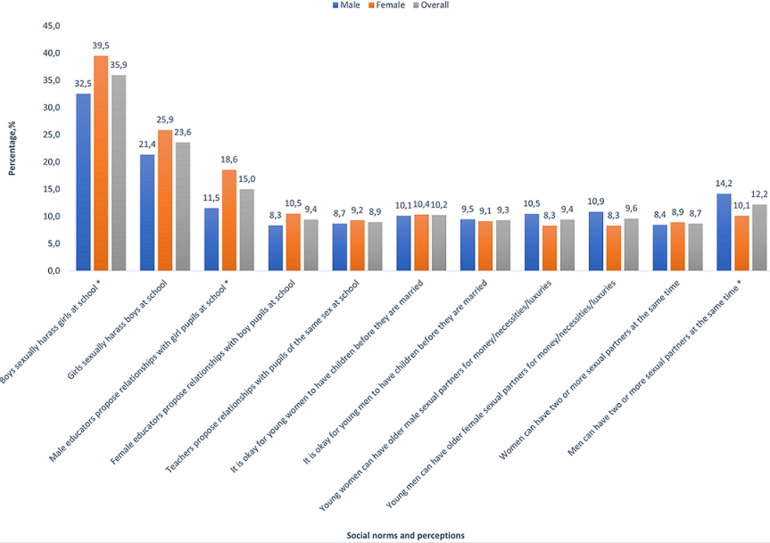
social norms and perceptions at the intersection of gender and sexual intimacy among South African adolescents aged 15-18 years, 2017/2018

**Social norms related to HIV, gender, and sexual intimacy:** overall, 29.3% reported ever having sexual intercourse, of which 72.5% reported past-year sexual activity. Of those sexually active in the past year, 64.6% regularly used condoms and 18.8% had multiple sex partners, with more males (25.4%) than females (10.4%, p=0.002) reporting multiple sex partners. Compared to their female counterparts, a significantly higher percentage of male adolescents endorsed the statement “men can have two or more sexual partners at the same time” (14.2% vs 10.1%, p=0.021). Only 8.4% of males (females, 8.9%, p=0.761) however endorsed the statement “women can have two or more sexual partners at the same time” (Figure 1). More males than females were cited as instigators of inappropriate sexual encounters within the school environment-male teachers, and male students alike. For example, the overall percentage reporting that their teachers proposed romantic relationships with students of the opposite gender was higher in relation to male teachers (11.5%) than female teachers (8.3%); 8.7% of participants reported that their teachers were romantically involved with students of the same sex. When “boy pupils” were the targets of inappropriate sexual encounters with female teachers, no difference existed between male and female adolescent participants´ report of such encounters (p=0.166). However, in situations where “girl pupils” were the targets of inappropriate sexual encounters with male teachers, report of such encounters by male adolescent participants (11.5%) was significantly lower than their female counterparts (18.6%, p<0.001). Within peer-to-peer relationships as well, girls were more common victims of sexual harassment; overall, 32.5% of participants reported that “boys sexually harass girls by touching, threatening or making rude remarks to them” vs 21.4% who reported the reverse. About 1 in 10 of adolescents overall (with no significant gender differences) were in favour of “sugar daddies” for young women (9.4%), “sugar mamas” for young men (9.6%), as well as having children out of wedlock for young women (10.2%) and young men (9.3%).

**Figure 2:**
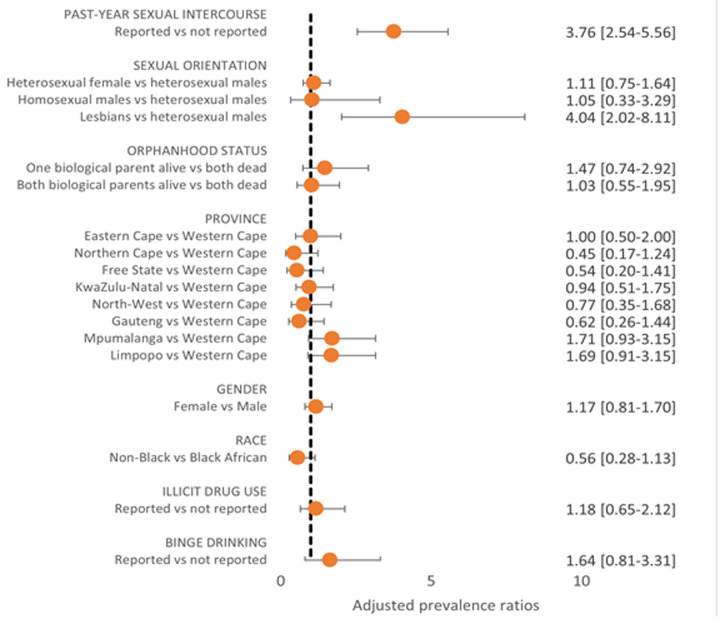
factors associated with perceived HIV risk among seronegative youth aged 15-18 years in South Africa, 2017/2018 (n = 2,896)

**Figure 3:**
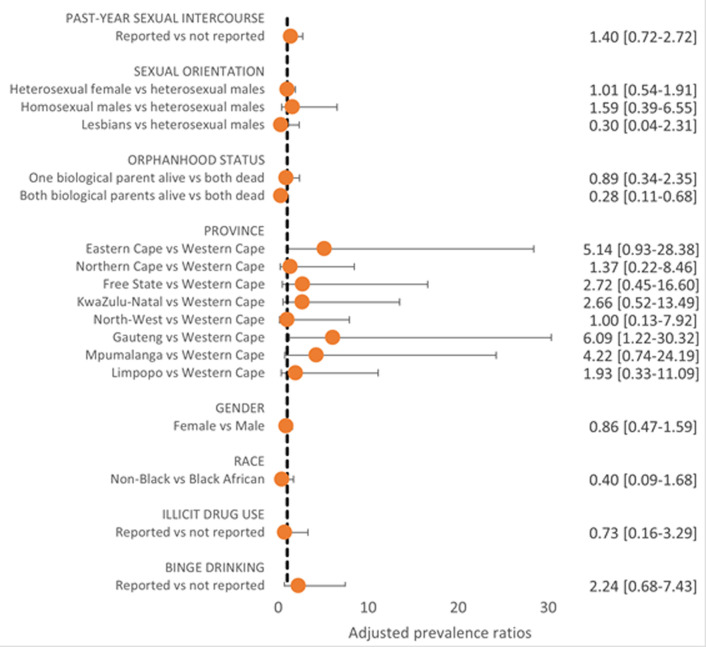
factors associated with HIV seropositivity among all surveyed adolescents aged 15-18 years, South Africa, 2017/2018 (n = 4,567)

**Discordance between actual vs perceived HIV risk:** HIV seropositivity overall was 4.5%. Of seronegative individuals, 10.1% perceived themselves at high risk for HIV. Within bivariate analyses, perceived risk differed significantly by past-year sexual activity, province, and sexual orientation, but not by gender, race, substance use, binge drinking, or orphanhood status (Figure 2). Perceived risk was higher among those reporting than not reporting past-year sexual activity (22.4% vs 6.0%, p<0.001). Perceived risk within provinces was: Northern Cape (4.6%), Free State (5.5%), Gauteng (6.1%), North-West (7.6%), KwaZulu-Natal (9.8%), Western Cape (10.2%), Eastern Cape (10.2%), Limpopo (16.2%), and Mpumalanga (17.8%). Discordance in perceived vs actual risk was seen by province; three of the five provinces with lowest perceived HIV risk reported the highest seroprevalence (KwaZulu-Natal, Free State, and Gauteng). Lesbians perceived the highest perceived risk for HIV (40.9%), compared to heterosexual males (9.2%), heterosexual females (10.3%), and homosexual/bisexual males (10.8%). While past-year sexual activity (APR=3.76, 95%CI, 2.54-5.56) and identifying as a lesbian compared to being a heterosexual male (APR=4.04, 95%CI, 2.02-8.11) were both strong predictors of perceived HIV risk after adjusting for age and sex, neither were significantly associated with HIV seropositivity among this adolescent population. Differences in perceived vs actual risk by province within adjusted analysis are shown in Figure 2 and Figure 3.

**Figure 4:**
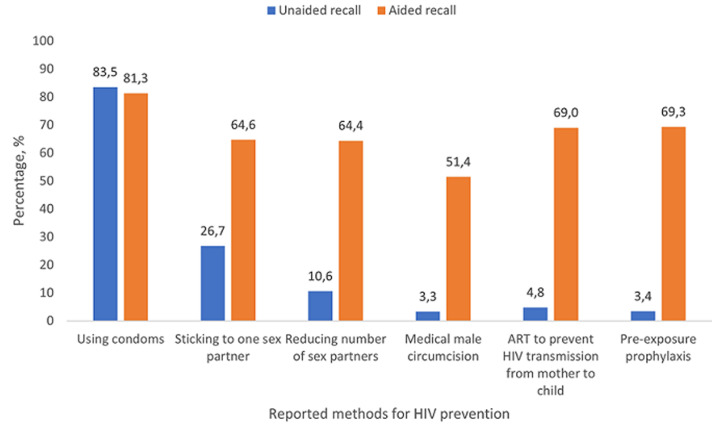
reported methods for HIV prevention based on unaided and aided recall among South African adolescents aged 15-18 years, 2017/2018

**Figure 5:**
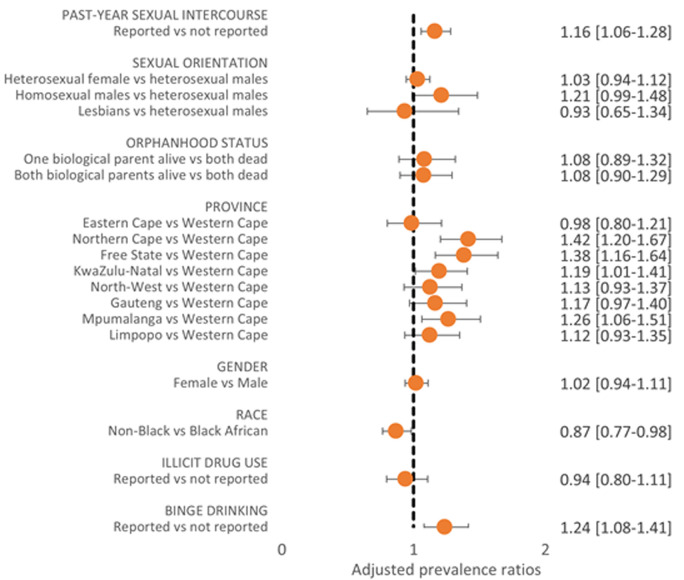
factors associated with openness to using PrEP among seronegative adolescents aged 15-18 years, South Africa, 2017/2018

**Prevalence and correlates of awareness of PrEP and other HIV preventive measures:** only a third of participants (33.2%) reported there were enough community-based organizations helping with HIV/AIDS in their community, and it was particularly low in Eastern Cape (19.3%). Awareness of HIV campaigns was high (95.5%) but past engagement was low (17.5%). Overall, 66.9% perceived that parents are discussing HIV prevention with their children (including condom use) (Table 1). Based on unaided recall, the most widely known HIV preventive measure was condom use (83.5%), very similar to the aided recall (81.3%) (Figure 4). Much fewer adolescents however spontaneously reported the following measures: sticking to one sexual partner (26.7% vs aided recall, 64.6%), reducing the number of sexual partners (10.6%, vs aided recall, 64.4%), medical male circumcision (3.3%, vs aided recall, 51.4%), and ART to prevent vertical transmission (4.8%, vs aided recall, 69.0%). Even with aided recall, large differences existed among population subgroups in awareness of these preventive measures as shown in Table 1. For example, awareness was generally high in KwaZulu-Natal, especially for condom use (87.5%) and medical male circumcision (66.3%); in contrast, awareness of medical circumcision was lowest in Limpopo (32.8%), while awareness of sticking to one sexual partner as a HIV preventive measure was lowest in Mpumalanga (54.1%). Based on unaided recall, only 3.7% overall reported PrEP (3.4% among seronegative adolescents) as an HIV preventive measure. On learning about PrEP, however, 69.3% of seronegative adolescents were open to using it. Openness towards PrEP was significantly higher among those reporting vs not reporting past-year sexual activity (APR=1.16, 95%CI, 1.06-1.28), and binge drinking (APR=1.24, 95%CI, 1.08-1.41). Compared to those living in Western Cape, openness to PrEP was significantly higher among those living in Northern Cape (APR=1.42, 95%CI, 1.20-1.67), Free State (APR=1.38, 95%CI, 1.16-1.64), KwaZulu-Natal (APR=1.19, 95%CI, 1.01-1.41), and Mpumalanga (APR=1.26, 95%CI, 1.06-1.51) (Figure 5).

**Table 1:** attitudes and perceptions towards HIV prevention interventions and programs among adolescents aged 15-18 years, South Africa, 2017/2018

	Interventions perceived to be effective at preventing HIV, % (95% CI)				Perceptions towards family and community programs for HIV prevention, % (95% CI)			
Characteristics	Aware of effectiveness of using condoms (n=4,567)	Aware of effectiveness of medical male Circumcision (n=4,567)	Aware of effectiveness of sticking to one sex partner (n=4,567)	Open to using (among only HIV seronegative individuals, n=2,896)	There are enough community-based organizations helping with HIV/AIDS in your community (n=4,567)	Perceive parents are discussing HIV prevention with their children (including condom use, n=4,567)	Aware of any community HIV prevention program (n=4,567)	Reported participation in any community HIV program (n=4,567)
**Total**	81.3 (79.3-83.3)	51.4 (48.8-54.0)	64.6 (62.2-67.1)	69.3 (66.3-72.3)	33.2 (30.8-35.6)	66.9 (64.5-69.3)	95.5 (94.5-96.6)	17.5 (15.4-19.5)
**Province**								
Western Cape	81.4 (75.5-87.3)	36.5 (29.7-43.4)	73.9 (67.6-80.3)	60.3 (51.6-68.9)	26.5 (20.3-32.7)	75.4 (69.5-81.4)	93.3 (88.6-98.0)	13.0 (8.2-17.9)
Eastern Cape	82.1 (76.5-87.7)	33.8 (26.7-40.9)	62.5 (55.3-69.8)	59.4 (50.5-68.3)	19.3 (13.2-25.3)	60.4 (53.1-67.6)	96.3 (93.5-99.1)	13.7 (8.3-19.1)
Northern Cape	79.2 (71.7-86.7)	36.4 (28.2-44.6)	65.1 (56.4-73.7)	85.3 (78.8-91.9)	42.7 (34.2-51.2)	56.3 (47.7-64.8)	97.1 (94.6-99.6)	18.2 (11.1-25.3)
Free State	83.3 (76.6-90.1)	59.6 (50.8-68.5)	71.8 (63.7-79.8)	83.4 (75.8-91.0)	29.3 (21.1-37.5)	71.9 (63.6-80.2)	97.0 (94.0-100.0)	26.7 (18.7-34.7)
KwaZulu-Natal	87.5 (84.4-90.6)	66.3 (61.7-70.8)	73.3 (69.1-77.5)	72.3 (66.6-78.0)	28.4 (24.1-32.8)	68.3 (63.8-72.7)	93.5 (91.0-96.1)	13.3 (10.0-16.6)
North-West	67.4 (60.1-74.8)	58.0 (50.3-65.6)	56.8 (49.1-64.5)	67.6 (58.8-76.5)	24.9 (18.1-31.7)	65.1 (57.6-72.5)	93.6 (89.8-97.5)	22.4 (16.0-28.9)
Gauteng	80.9 (75.2-86.7)	55.5 (48.2-62.8)	59.2 (51.9-66.4)	69.9 (61.9-78.0)	39.2 (32.1-46.4)	69.7 (63.0-76.4)	98.0 (95.9-100.0)	28.3 (21.8-34.9)
Mpumalanga	77.2 (71.7-82.8)	59.2 (52.6-65.8)	63.7 (57.3-70.1)	76.5 (68.8-84.1)	51.3 (44.7-58.0)	70.3 (64.2-76.4)	94.1 (91.0-97.1)	11.0 (6.6-15.4)
Limpopo	79.2 (73.1-85.4)	32.8 (26.0-39.6)	54.1 (46.9-61.4)	67.2 (59.3-75.1)	39.1 (32.1-46.1)	55.5 (48.3-62.7)	97.3 (95.0-99.6)	10.4 (6.1-14.7)
**HIV status**								
Seronegative	80.8 (78.3-83.2)	49.4 (46.3-52.4)	63.9 (61.0-66.9)	N/A	32.9 (30.0-35.9)	66.7 (63.8-69.6)	96.0 (94.8-97.2)	18.0 (15.6-20.5)
Seropositive	69.8 (55.0-84.5)	59.9 (44.5-75.3)	57.5 (41.8-73.3)	N/A	42.3 (26.6-58.0)	65.5 (50.9-80.1)	98.0 (94.1-100.0)	25.4 (11.6-39.1)
**Geographic location**								
Urban	81.3 (78.5-84.0)	51.7 (48.1-55.2)	64.6 (61.2-68.0)	69.7 (65.7-73.8)	34.1 (30.8-37.5)	69.5 (66.3-72.7)	95.6 (94.2-97.1)	21.0 (18.1-24.0)
Rural informal	81.8 (78.9-84.8)	52.1 (48.2-56.0)	64.2 (60.4-67.9)	68.9 (64.4-73.3)	33.3 (29.6-36.9)	63.0 (59.2-66.7)	95.5 (93.9-97.1)	11.9 (9.4-14.3)
Rural (farms)	76.2 (68.2-84.1)	40.8 (31.4-50.1)	69.8 (61.7-78.0)	66.4 (54.4-78.5)	16.5 (10.5-22.6)	59.2 (49.7-68.8)	94.3 (90.9-97.8)	10.3 (4.8-15.8)
**Gender**								
Male	81.2 (78.3-84.1)	55.0 (51.3-58.6)	63.7 (60.1-67.2)	68.6 (64.3-73.0)	34.8 (31.3-38.3)	65.6 (62.1-69.0)	95.6 (94.2-97.1)	15.6 (12.8-18.4)
Female	81.4 (78.6-84.1)	47.7 (44.1-51.3)	65.7 (62.2-69.1)	70.0 (66.0-74.0)	31.6 (28.2-34.9)	68.2 (64.9-71.6)	95.5 (93.9-97.0)	19.4 (16.4-22.4)
**Race**								
Black African	81.6 (79.4-83.8)	53.1 (50.2-55.9)	62.4 (59.7-65.2)	70.8 (67.6-74.1)	34.7 (32.0-37.5)	66.0 (63.3-68.7)	96.4 (95.4-97.3)	18.0 (15.7-20.3)
Non-Black	79.7 (74.8-84.6)	42.7 (36.9-48.5)	76.2 (70.9-81.4)	61.5 (54.4-68.7)	25.3 (20.7-29.9)	71.3 (66.2-76.4)	91.4 (87.3-95.4)	14.8 (11.0-18.7)
**Sexual orientation**								
Heterosexual males	81.1 (78.1-84.1)	55.3 (51.6-59.0)	63.5 (59.8-67.1)	68.0 (63.6-72.5)	35.1 (31.5-38.7)	65.1 (61.6-68.6)	95.7 (94.2-97.2)	15.4 (12.6-18.2)
Heterosexual females	81.2 (78.3-84.0)	47.7 (44.1-51.4)	65.3 (61.8-68.8)	70.1 (66.0-74.2)	30.9 (27.5-34.3)	68.2 (64.8-71.6)	95.6 (94.0-97.1)	19.3 (16.3-22.3)
Homosexual males	85.7 (74.0-97.3)	45.4 (26.1-64.7)	70.0 (53.3-86.7)	83.9 (67.8-100.0)	26.1 (7.9-44.3)	80.9 (62.9-98.9)	93.4 (86.3-100.0)	20.4 (2.0-38.8)
Lesbians	88.3 (78.1-98.5)	48.2 (27.4-69.1)	79.1 (63.4-94.8)	64.0 (41.0-87.0)	56.4 (36.7-76.1)	71.6 (54.5-88.6)	91.3 (82.1-100.0)	21.8 (1.7-41.9)
**Orphanhood status**								
Both biological parents dead	80.2 (72.4-88.0)	47.9 (38.2-57.7)	62.5 (52.8-72.1)	65.0 (53.7-76.3)	39.7 (29.8-49.7)	67.0 (58.3-75.7)	96.9 (93.9-99.9)	25.2 (16.5-33.8)
One biological parent dead	79.3 (74.6-84.0)	53.2 (47.4-59.0)	63.0 (57.4-68.7)	69.8 (63.5-76.2)	32.0 (26.5-37.4)	62.1 (56.4-67.8)	96.5 (94.7-98.3)	18.2 (13.6-22.8)
Both biological parents alive	82.0 (79.6-84.3)	51.4 (48.3-54.4)	65.3 (62.4-68.2)	69.6 (66.1-73.1)	32.9 (30.1-35.8)	68.1 (65.3-70.9)	95.2 (93.9-96.5)	16.4 (14.1-18.8)
**Sexual intercourse**								
Never	79.5 (77.0-82.0)	50.7 (47.7-53.8)	63.6 (60.6-66.5)	65.9 (62.3-69.6)	30.2 (27.4-33.0)	64.8 (61.9-67.7)	94.1 (92.7-95.6)	15.7 (13.4-18.1)
Ever	85.7 (82.2-89.3)	53.4 (48.4-58.4)	66.2 (61.4-71.0)	76.0 (70.9-81.1)	39.4 (34.4-44.3)	71.6 (67.0-76.2)	99.4 (98.8-100.1)	21.8 (17.6-26.0)

**Proximal social influences at home, school, and the community:** being exposed to inappropriate sexual behaviours from teachers at school was associated with greater permissiveness towards risky sexual behaviours. For example, those who observed any form of romantic relationship between teachers and students in their school were less likely to report that “the risk of HIV transmission be reduced by having sex with only one uninfected partner who has no other partners” (APR=0.85, 95%CI, 0.73-0.99), or that “a person can reduce the risk of getting HIV by using a condom every time he/she has sex” (APR=0.87, 95%CI, 0.79-0.96). Conversely, perceived parental sex education in the home and presence of community educational programs were positively associated with increased likelihood of being aware of HIV preventive measures and being open to PrEP (Table 2). Perceived parental sex education was associated with higher likelihood of being aware that “a healthy-looking person can have HIV” (APR=1.13, 95%CI, 1.05-1.20), and that the risk of HIV can be reduced by having fewer sexual partners (APR=1.16), being faithful to one sex partner (APR=1.14), condom use (APR=1.14), and medical male circumcision (APR=1.28) (all p<0.05). Compared to those campaign-unaware, knowledge of HIV preventive measures was significantly higher among those campaign-aware and campaign-engaged, respectively, for the following: fewer sexual partners (APR=1.28 and 1.38, aware and engaged respectively), being faithful to one sex partner (APR=1.39 and 1.62), condom use (APR=1.38 and 1.45), and medical male circumcision (APR=1.58 and 1.74 respectively) (all p<0.05).

**Table 2:** associations between proximal social influences and attitudes towards HIV prevention among adolescents aged 15-18 years, South Africa, 2017/2018

Exposure variable	Comparison groups	Outcome	Adjusted prevalence ratios, APR (95% CI)	P-value
Perceived parental sex education	Reported vs not reported	A person can reduce the risk of HIV by having fewer sexual partners	1.16 (1.05-1.30)	0.005
	Reported vs not reported	A healthy-looking person can have HIV	1.13 (1.05-1.20)	0.001
	Reported vs not reported	The risk of HIV transmission be reduced by having sex with only one uninfected partner who has no other partners	1.14 (1.03-1.27)	0.013
	Reported vs not reported	A person can reduce the risk of getting HIV by using a condom every time he/she has sex	1.14 (1.06-1.22)	<0.001
	Reported vs not reported	Medical male circumcision can reduce the risk of HIV infection in males	1.28 (1.12-1.47)	<0.001
	Reported vs not reported	Open to using PrEP (among HIV seronegative individuals)	1.16 (1.05-1.29)	0.003
Exposure to school environment where teachers have sexual relationships with students	Reported vs not reported	A person can reduce the risk of HIV by having fewer sexual partners	0.99 (0.87-1.13)	0.897
	Reported vs not reported	A healthy-looking person can have HIV	1.03 (0.96-1.10)	0.450
	Reported vs not reported	The risk of HIV transmission be reduced by having sex with only one uninfected partner who has no other partners	0.85 (0.73-0.99)	0.039
	Reported vs not reported	A person can reduce the risk of getting HIV by using a condom every time he/she has sex	0.87 (0.79-0.96)	0.008
	Reported vs not reported	Medical male circumcision can reduce the risk of HIV infection in males	1.00 (0.84-1.19)	0.996
	Reported vs not reported	Open to using PrEP (among HIV seronegative individuals)	0.96 (0.85-1.08)	0.508
Exposure to community education programs on HIV prevention	Aware only vs not aware	A person can reduce the risk of HIV by having fewer sexual partners	1.28 (0.96-1.71)	0.089
	Past active engagement vs not aware		1.38 (1.02-1.86)	0.038
	Aware only vs not aware	A healthy-looking person can have HIV	1.34 (1.07-1.68)	0.010
	Past active engagement vs not aware		1.43 (1.14-1.80)	0.002
	Aware only vs not aware	The risk of HIV transmission be reduced by having sex with only one uninfected partner who has no other partners	1.39 (1.02-1.89)	0.036
	Past active engagement vs not aware		1.62 (1.18-2.22)	0.003
	Aware only vs not aware	A person can reduce the risk of getting HIV by using a condom every time he/she has sex	1.38 (1.08-1.77)	0.011
	Past active engagement vs not aware		1.45 (1.12-1.87)	0.004
	Aware only vs not aware	Medical male circumcision can reduce the risk of HIV infection in males	1.58 (1.03-2.41)	0.036
	Past active engagement vs not aware		1.74 (1.12-2.71)	0.014
	Aware only vs not aware	Open to using PrEP (among HIV seronegative individuals)	1.66 (1.17-2.36)	0.005
	Past active engagement vs not aware		1.76 (1.23-2.52)	0.002

## Discussion

Our results suggest that the seeds of the imbalanced societal gender structures that drive the HIV epidemic among adults may be planted in adolescence. Girls were more likely to be sexually harassed by male peers and male teachers, than the reverse. Also, male adolescents were a lot more permissive towards young men having multiple female sexual partners than with the reverse. Further evidence of the normalizing of such patriarchal values in adolescence is suggested from the observation that while female participants were as likely as their male counterparts to notice (and report) instances of 'boy pupils' being sexually involved with female teachers, male adolescents reported significantly lower prevalence than their female counterparts for similar occurrences involving 'girl pupils' and male teachers. This could suggest either that such occurrences occur in secret (possibly explaining the higher burden of internalized stigma among females living with HIV), or that they are non-secret encounters but normalized, perhaps by their perceived frequency (descriptive social norms), or their perceived social acceptability (injunctive social norms). Besides the risk of HIV transmission, such harmful social norms may erode decades of progress from population-level HIV prevention campaigns. For example, we found that youth exposed to any form of inappropriate sexual behavior from school were more dismissive of using condoms and of having fewer sexual partners as strategies for HIV prevention. Intensified efforts are needed to shift social norms in these areas; our findings warrant this. Unaided recall, which is a much stronger predictor of behavior than aided recall [12], was very low for several of the evidence-based interventions except condom use which had a very high unaided recall (83.5%). However, we observed dissonance between what adolescents knew or believed, versus their behaviors. For example, despite the high unaided recall for condom use as an HIV prevention measure, over a third (35.4%) of those who reported past-year sexual activity indicated they did not use condoms frequently. This cognitive-behavioral dissonance suggests that knowledge alone is not sufficient for behavior change and should be accompanied by interventions that seek to recalibrate social norms among youth. Unaided recall of PrEP was very low in our study (3.7%), but most of those who were HIV seronegative (69.3%) were open to using it after learning about it.

Openness to PrEP was higher among those reporting past-year sexual activity and binge-drinking, which may reflect perceived risk for HIV infection. No significant difference in PrEP openness existed by gender, despite women being the single most affected demographic group for HIV in South Africa. Targeted interventions may be needed to promote adoption of evidence-based preventive interventions among those at highest risk to mitigate future burden of disease. While adult HIV infections are overwhelmingly sexually related, new cases in the pediatric population are predominantly from vertical transmission [13] which might explain why sexual activity was not significantly associated with HIV seropositivity in our study. However, as adolescents transition to adults, their risk factors might change, possibly explaining why seronegative individuals reporting past-year sexual activity perceived themselves to be at high risk for HIV. Among certain subgroups, we observed perceived risk for HIV that was incongruent with actual risk. For example, self-rated HIV risk among individuals identifying as lesbians was four-fold higher compared to heterosexual males, an observation that is inconsistent with real-world risk profiles. The actual concern is not overestimation of risk per se, but underestimation, as this might drive more risk-taking behavior. For example, driven by the misperception that male medical circumcision offers 100% protection against HIV, as has been well documented [14-16], some heterosexual men may practice condomless sex, thus increasing their risk for HIV infection. Only one third of participants (33.2%) of participants reported there were enough community-based organizations in their local community for HIV prevention, which might explain why actual engagement with HIV prevention organizations was low (17.5% overall). With the explosion in the popularity and youth engagement with social media such as Tiktok, opportunities exist to expand the reach of traditional campaigns [17,18]. Such youth-led modern campaigns may foster a sense of ownership, give youth a voice to share things in the virtual space that they may otherwise not feel comfortable sharing in person, and may help to reduce stigma. More research is needed in this domain.

**Strengths and Limitations:** the strength of this study is the use of a nationally representative sample of South African youth to examine issues of significant public health interest in the South African context. HIV status was objectively ascertained which increases the internal validity. Nonetheless, this study has certain limitations. First, the sampling frame excluded school dormitories, nursing homes, hospitals, homeless shelters, and military barracks; these results may not be generalizable to these settings. Similarly, the results from this study may not be generalizable to youth and young adults outside of the studied age bracket of 15-18 years. Second, these are cross-sectional data and only associations can be drawn. Third, some of the definitions may be subject to misclassification; for example, a history of regular condom use was defined as a report of using condoms “every time” with at least one of the past three sexual partners. This may however not necessarily indicate the individual used a condom during their last sexual encounter. Finally, self-reported data may be subject to misclassification.

## Conclusion

Female teens were more likely to be sexually harassed at school, and male teens were a lot more permissive towards young men having multiple female sexual partners than with the reverse. Condoms were the most popular method of HIV prevention, with 83.5% of all participants reporting awareness. Yet, over a third of those sexually active in the past year reported not using condoms all the time. Perceived parental sex education and presence of community campaigns for HIV prevention were both associated with increased awareness of HIV prevention measures as well as openness to PrEP among those who were seronegative. Unaided recall of PrEP was very low (3.7%), but most of those who were HIV seronegative (69.3%) where open to using it after learning about it. Opportunities exist to educate and increase barrier-free access to evidence-based preventive strategies.

### What is known about this topic

- In 2016, it was reported that adolescent girls and young women aged 15–24 years accounted for 37% of new infections;
- Less than half of sexually active adolescents aged 14-17 years in rural South Africa reported a history of regular condom use (45.5% boys; 46.5% girls), and less than half (44.7%) of South African young adults aged 18-24 years from two high HIV prevalence districts had correct knowledge of HIV prevention.

### What this study adds

- Even among this adolescent population, we found biased perceptions of gender norms and roles; compared to their female counterparts, a significantly higher percentage of male adolescents endorsed the statement “Men can have two or more sexual partners at the same time” (14.2% vs 10.1%, p=0.021);
- Condoms were the most popular method of HIV prevention, with 83.5% of all participants reporting awareness. Yet, 35.4% of those sexually active in the past year reported not using condoms all the time;
- Of HIV seronegative adolescents, 69.3% were interested in using pre-exposure prophylaxis on learning about it, though initial awareness was low.
